# Breast cancer with an intraductal component that was proven genetically to be metastasis of contralateral breast cancer: a case report

**DOI:** 10.1186/s40792-020-00966-y

**Published:** 2020-08-24

**Authors:** Yoshiaki Shinden, Hazuki Saho, Yuki Nomoto, Ayako Nagata, Koji Minami, Akihiro Nakajo, Toshiaki Akahane, Tsubasa Hiraki, Akihide Tanimoto, Tetsuhiro Owaki, Yuko Kijima, Shoji Natsugoe

**Affiliations:** 1 Department of Digestive Surgery, Breast and Thyroid Surgery, Kagoshima University Graduate School of Medical and Dental Sciences, 8-35-1, Sakuragaoka, Kagoshima, 890-8520 Japan; 2 Department of Pathology, Kagoshima University Graduate School of Medical and Dental Sciences, Kagoshima, Japan; 3 Education Center for Doctors in Remote Islands and Rural Areas, Kagoshima University Graduate School of Medical and Dental Sciences, Kagoshima, Japan; 4 Department of Breast Surgery, School of Medicine, Fujita Health University, Toyoake, Japan

**Keywords:** Bilateral breast cancer, Metastatic breast lesion, Intraductal components

## Abstract

**Background:**

When diagnosing patients with bilateral breast cancer, it is challenging to determine the relationship between multiple breast cancer lesions at the individual patient level with certainty.

**Case presentation:**

A 35-year-old Japanese woman was diagnosed with a left breast cancer. She was previously diagnosed with right pT3N3M0 stage IIIC breast cancer and underwent chemotherapy with targeted therapy, radiotherapy, and endocrine therapy as adjuvant treatment after mastectomy and axillary lymph node dissection. Approximately 2 years after the first surgery, her left breast cancer was preoperatively diagnosed as a contralateral primary breast cancer, and left mastectomy and axillary lymph node dissection were performed. Histopathologically, the tumor was determined to be invasive ductal carcinoma accompanied with several intraductal components. After a second surgery, mutation analysis of her bilateral breast cancer was performed in a clinical study, which revealed that her metachronous bilateral breast tumors had the same *GATA3* and *CSMD1* mutations. Thus, mutation analysis strongly supported her latter left breast cancer being a metastatic lesion from the former right breast cancer. Some difficulties in diagnosing bilateral breast cancer exist when determining whether they are double primary cancers or represent contralateral breast metastasis. The existence of intraductal components is a critical piece of information for suspecting primary lesions. However, this case demonstrated that metastatic contralateral breast lesions can have intraductal components.

**Conclusion:**

Herein we report a genetically proven contralateral breast metastasis with some intraductal components.

## Background

Breast cancer is the most frequently diagnosed cancer and the leading cause of cancer death in females worldwide [1]. Among breast cancer patients, 2 to 11% have bilateral breast cancer [2–5]. When diagnosing patients with bilateral breast cancer, it is critical to determine whether they have bilateral primary cancers or metastatic contralateral breast cancer, because the treatment strategies differ. Several diagnostic criteria exist for bilateral breast cancer, but it is challenging to determine the relationship between multiple breast cancer lesions at the individual patient level with certainty [6].

Recently, cancer genomics have evolved at both the preclinical and clinical levels. Furthermore, several studies have used genomic sequencing to analyze bilateral breast cancer [7, 8]. Herein we report a case of metachronous bilateral breast cancer in whom the second breast cancer was diagnosed as a metastatic lesion from contralateral breast cancer using mutation analysis.

## Case presentation

**Fig. 1:**
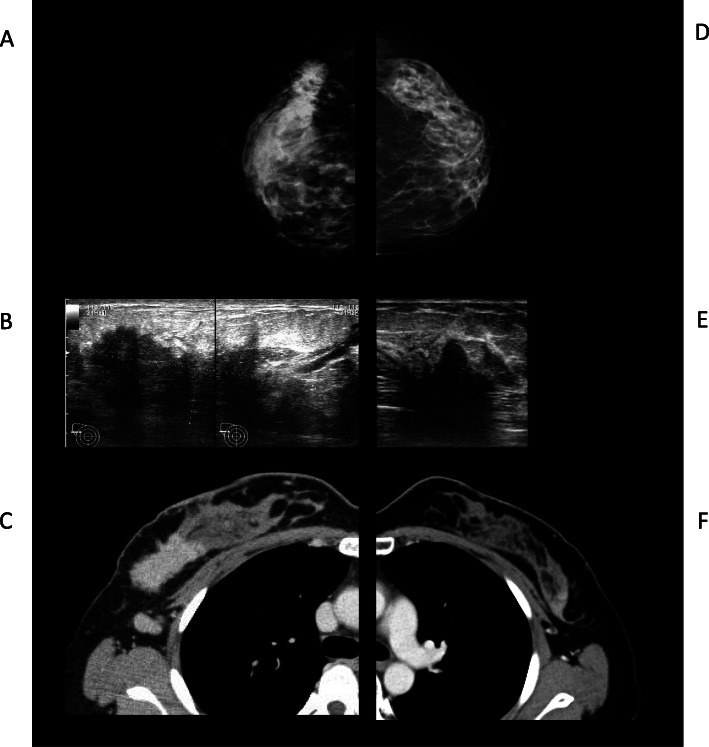
Preoperative imaging of right and left breast lesions. Mammography (cranio-caudal view) revealed a mass in the middle outer portion of the right breast (**a**). No lesions were detected in the left breast on mammography (**d**). Ultrasonography showed a 59-mm hypoechoic mass with an unclear margin in the right breast (**b**), and an 18-mm hypoechoic lesion with an unclear margin in the left breast (**e**). Computed tomography (CT) with contrast revealed a 53-mm mass in the right breast (**c**). Several axillary lymph node metastases were detected, but no other metastasis, on CT. In the left breast, CT with contrast demonstrated a 34-mm mass but no other metastatic lesions (**f**)

A 35-year-old Japanese woman presented with a hypoechoic mass in her left breast. She had been diagnosed with right breast cancer 2 years ago and underwent right mastectomy and axillary lymph node resection. This cancer was diagnosed as pT3N3M0 stage IIIC, luminal-HER2 (ER-positive, PgR-positive, HER2-positive, and Ki-67 index 35.6%), and she underwent chemotherapy with targeted therapy (docetaxel, cyclophosphamide, and trastuzumab), radiotherapy (to the chest wall and axillary region), and endocrine therapy (tamoxifen and leuprorelin) as adjuvant treatment. Twenty-three months after finishing trastuzumab and 28 months into endocrine therapy, a 1.8 × 0.6 cm irregular hypoechoic mass was detected in the upper outer region of her left breast. Preoperative findings by imaging modalities are shown in Fig. 1. With core needle biopsy, the left breast mass was diagnosed pathologically as invasive ductal carcinoma. No additional lesions were observed on mammography. Computed tomography and bone scanning showed no evidence of distant metastasis. The left breast cancer was preoperatively diagnosed as a contralateral primary breast cancer as T1N0M0 stage IA, and left mastectomy and sentinel lymph node biopsy were performed. Since a macrometastasis was found in the sentinel lymph node during intraoperative pathological diagnosis, axillary lymph node dissection was added.

**Fig. 2:**
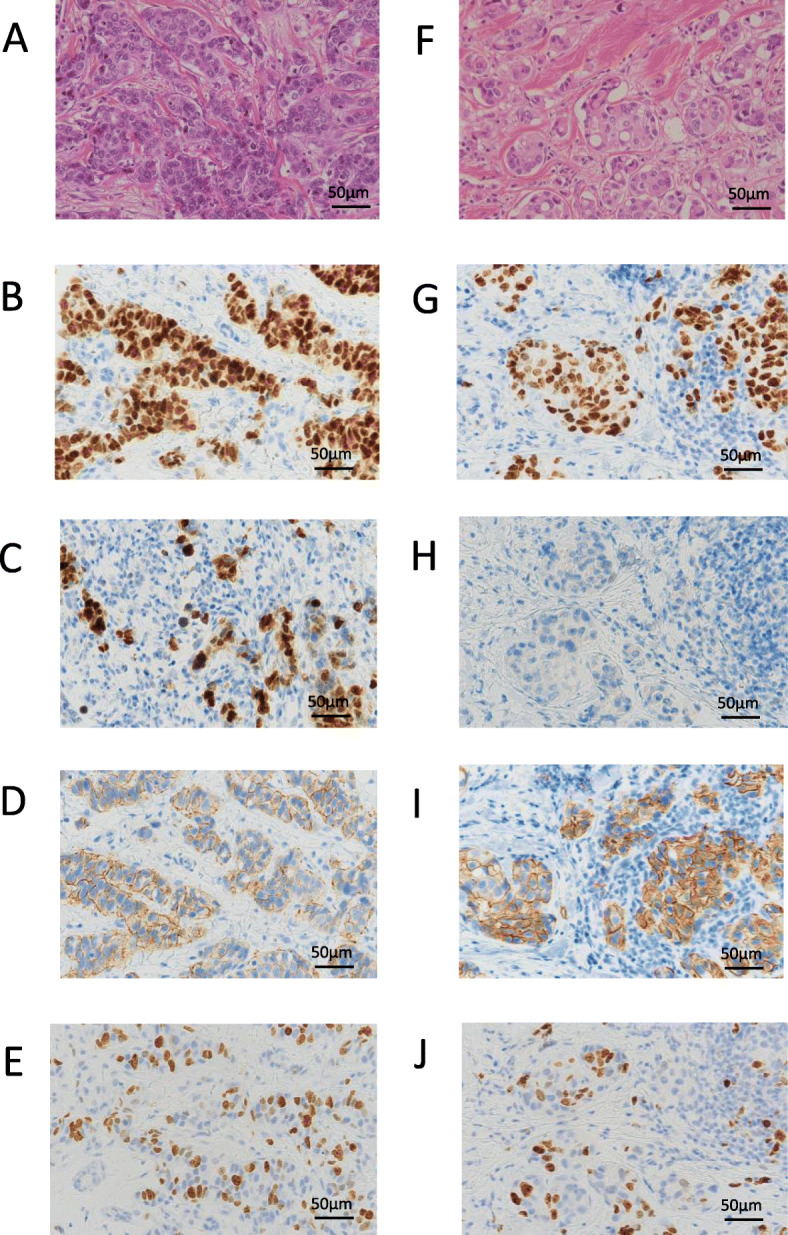
Histopathological findings of bilateral breast cancers. The right breast cancer is shown in **a**–**e**, and the left breast cancer is shown in **f**–**j**: hematoxylin-eosin staining (**a**, **f**), ER (**b**, **g**), PgR (**c**, **h**), HER2 (**d**, **i**), and Ki-67 (**e**, **j**). Immunohistochemical staining results were ER, 5; PgR, 4; HER2, 2+; and Ki-67, 35.6% for the right lesion, and ER, 5; PgR, 1; HER2, 2+; and Ki-67, 20% for the left lesion

**Fig. 3:**
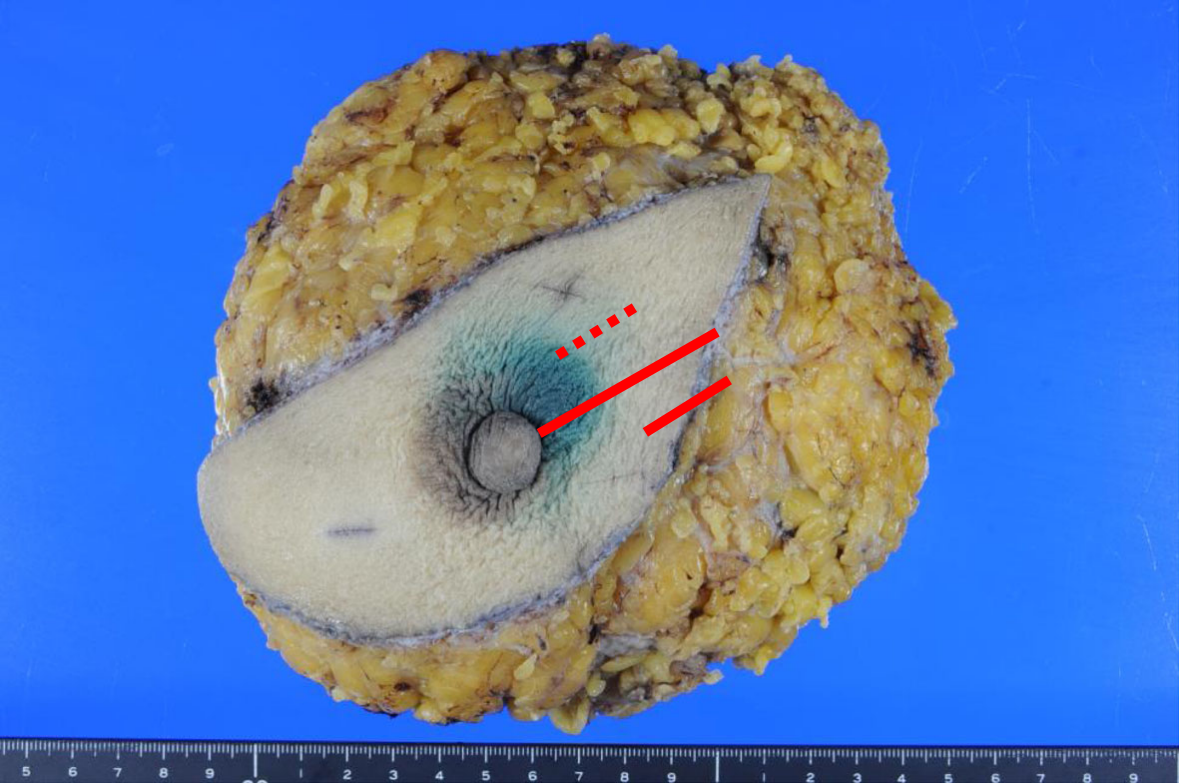
Macroscopic distribution of cancer in the left breast. A few intraductal components were present (dotted red line) in part of the invasive area (red line)

**Fig. 4:**
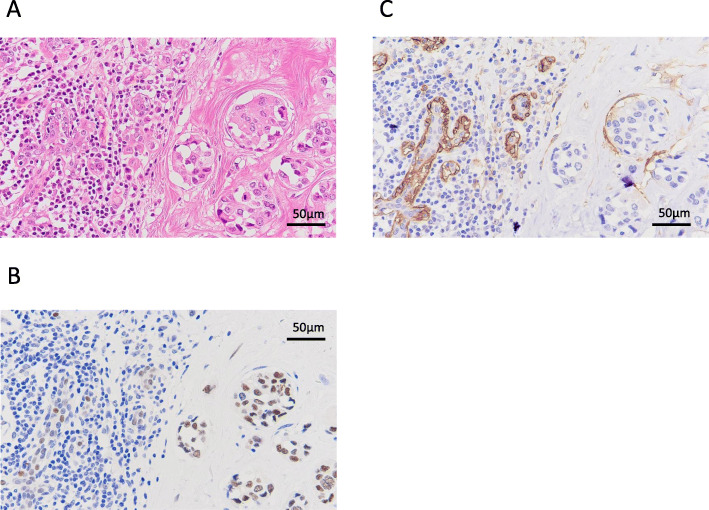
Intraductal metastatic carcinoma lesion in the left breast. Hematoxylin-eosin staining (**a**), ER (**b**), and CD10 (**c**)

Histologically, the tumor was an invasive ductal carcinoma with 4.8 × 2.0 cm in size. Several intraductal components and lymphatic invasion were observed. The stage was determined to be pT2N1M0 (stage IIB). Immunohistochemical examination revealed that the tumor was ER-positive, PgR-negative, and HER2-positive, with a Ki-67 index of 20% (Fig. 2). Although bilateral breast cancer subtypes were similar, eventually, we judged the left breast cancer to be a second primary lesion as the reason for existence because of the intraductal components (Figs. 3 and 4). Postoperatively, chemotherapy and targeted therapy (docetaxel, trastuzumab, and pertuzumab) and endocrine therapy (toremifene and leuprorelin) were administered.

After surgery, mutation analysis for her bilateral breast cancer was performed as part of a clinical study. The study was approved by the institutional review board of Kagoshima University Hospital, and informed consent was acquired.

DNA was extracted from FFPE samples from the resected breast tumors, a residual liquid-based cytology (LBC) sample from preoperative biopsy examination, and blood. For the FFPE and LBC samples, DNA extraction was performed with a Maxwell 16 FFPE Tissue LEV DNA Purification Kit (Promega, Madison, WI, USA). For the blood sample, DNA extraction was performed with the Maxwell RSC Blood DNA Kit (Promega). The procedures were conducted according to the manufacturer’s instructions. Extracted DNA was sequenced according to the QIAGEN breast cancer panel protocol, which contains 93 genes. Using germline mutation analysis with the blood sample as a reference, only somatic mutations in tumor samples were analyzed using a web portal. This analysis revealed that her metachronous bilateral breast tumors had the same *GATA3* and *CSMD1* mutations (Table 1). These results strongly suggested that her latter left breast cancer was a metastatic lesion from the former right breast cancer. No other mutations were detected. The copy numbers of ERBB2 were increased by the same degree in both lesions. Adjuvant therapy has been continued, and no recurrence has occurred in the 12 months after her second surgery.

**Table 1:** Somatic mutations in tumor samples

	Right FFPE	Left FFPE	Left LBC
GATA3	p.Ser437fs*>9 vaf 18%	p.Ser437fs*>9 vaf 18%	p.Ser437fs*>9 vaf 27%
CSMD1	p.Gly209Arg vaf 14%	p.Gly209Arg vaf 16%	p.Gly209Arg vaf 21%

We experienced a genetically proven contralateral breast metastasis that had some intraductal components. When we diagnose bilateral breast cancers, the question of whether the contralateral breast lesion is primary or metastatic always arises. Robbins and Berg defined the following criteria for metastatic breast lesions: first, metastases are more likely to be near the midline or in the fatty tail; second, multiple metastases are present; third, spread occurs in an expansive fashion; and fourth, metastases are not associated with contiguous in situ carcinoma [3]. Additional criteria for metastatic breast lesions include the presence of distant metastasis, the existence of lesions in the fat surrounding the breast parenchyma, the histological similarity to the primary lesion, and a short time interval between times of tumor onset [6, 9]. We diagnose bilateral breast cancer cases considering all these factors clinically and pathologically. In particular, demonstration of in situ carcinoma contiguous to the invasive carcinoma is regarded as a critical factor for diagnosing a lesion as a primary breast cancer [9, 10].

In contrast, previous studies that analyzed bilateral breast cancer using karyotypic profiles or allelic imbalances demonstrated that metastatic contralateral breast cancer can have intraductal components [11]. Furthermore, the study authors stated that in situ lesions could no longer be considered as a criterion for de novo carcinogenesis.

Extensive intraductal component is reported to be more frequent in overexpressing HER2 tumors than luminal A tumors. This case was HER2 overexpressing tumor, and it might affect the existence of intraductal component in contralateral breast metastasis [12].

The present case had metachronous bilateral breast cancer. Clinically, whether her latter left breast cancer was primary or metastatic was controversial. The left lesion had the similar histological findings, ER status positivity, and HER2 expression as the right lesion. However, the left lesion had a different PgR status, was located in the outer upper region of the breast and far from the midline, and was not accompanied by distant metastatic lesions. Eventually, we diagnosed the latter breast cancer as a second primary lesion, because we detected in situ carcinoma contiguous to the invasive carcinoma in this lesion. However, mutation analysis confirmed that her latter left breast lesion was a metastasis from her former right breast cancer.

Interestingly, in the present case, genetic mutation analysis results from the resected specimen and the preoperative LBC specimen matched. Currently, genetic evaluation is widely used; therefore, the efficacy and feasibility of genetic analysis for the diagnosis of bilateral breast cancer are improving. Less invasive examination techniques for genetic-based tumor diagnosis is demanding. Akahane et al. reported that LBC tumor specimens were of sufficient quality for use in next-generation sequencing (NGS) [13]. In the present case, we had acquired an LBC sample 10 months before DNA extraction, and the DNA quality was sufficient for NGS. In the future, we expect that NGS using preserved LBC specimens to analyze the mutation status of metastatic lesions less invasively will be increasingly used. If we could diagnose her second breast cancer as metastatic lesions without surgery, we might avoid the second surgery. Further clinical studies are needed.

## Conclusion

We have reported a case of metachronous bilateral breast cancer. Despite the left breast cancer having an intraductal component, mutation analysis suggested it was a metastatic lesion from the right breast cancer. Metastatic breast lesions can have intraductal components; thus, genetic analysis is important in the diagnosis of bilateral breast cancer.

## Data Availability

The data are not available for public access because of patient privacy concerns.

## Abbreviations

GATA3: GATA binding protein 3
CSMD1: CUB and Sushi multiple domains 1
ER: Estrogen receptor
PgR: Progesterone receptor
HER2(ERBB2): Human epidermal growth factor receptor 2
FFPE: Formalin fixed paraffin embedded
LBC: Liquid-based cytology
NGS: Next-generation sequencing
